# COVID-19-Combating English with Chinese Characteristics in the CAT’s Translations

**DOI:** 10.1177/21582440221124457

**Published:** 2022-09-24

**Authors:** Ping Li, Chuanmao Tian

**Affiliations:** 1 Qufu Normal University, Qufu, China; 2 Yangtze University, Jingzhou, China

**Keywords:** COVID-19, Chinese-English translation, China English, features, literal translation

## Abstract

During the COVID-19 outbreak, the China Academy of Translation translated and officially released five groups of *Key Words to Understand China: The Fight Against COVID-19* for daily precautions, policy publicity, and information dissemination. Based on the translations and relevant data from Chinese and English media, this study makes a tentative analysis of the linguistic, ideological, and cultural features of English used in the translations. The findings are that China English is outstanding with respect to translating some Chinese-specific words and expressions in the translations, which generally results from the method of literal rendering. It is suggested that literal translation and transliteration can be used more frequently in English texts related to big events in order to strengthen the Chinese characteristics of China English as a major variety of world Englishes.

## Introduction

COVID-19, or the novel coronavirus pneumonia, broke out on a large scale in Wuhan, China in January 2020, swept very fast across the whole world, and became a very severe Public Health Emergency of International Concern (PHEIC). Although the origin of COVID-19 has not yet been confirmed, the virus caused by SARS-CoV-2 instead of viral escape from a laboratory “most likely arose in bats, and then spread to humans via an as-yet unidentified intermediary animal,” according to the World Health Organization (WHO).^1^ In 3 months, more than 80,000 confirmed cases in China and more than two million infections around the world were reported in the middle of April 2020 (The World Health Organization [WHO], 2020). In response to the epidemic, various language services were provided in China in order to facilitate the communication between patients and health workers, or help foreigners to deal with and know about the outbreak in the country. For example, the Anti-coronavirus Language Service Corps (战疫语言服务团, *Zhànyì Yŭyán Fúwùtuán*) which was made up of language experts from a few famous universities, such as Beijing Language and Culture University, Central China Normal University, Wuhan University, and Tsinghua University, provided Chinese people and foreigners with two handbooks respectively, namely, *A Guide to the Prevention and Control of COVID-19 in Hubei Dialects* and *A Guide to the Prevention and Control of COVID-19 in Foreign Languages* (The Ministry of Education [MOE], 2020a). The latter contains common bilingual expressions on the COVID-19 prevention and treatment between Chinese and a dozen foreign languages, such as Japanese, Korean, Persian, Italian, Arabic, and English. The China Academy of Translation (CAT) which is affiliated to the China Foreign Languages Publishing Administration (CFLPA), a national foreign publicity department of the Central Government, had officially released three groups of epidemic-related translations by the end of March 2020 in order to help the international community to know about China’s fight against the coronavirus and provide reference for stopping the pandemic in other countries and regions (China Academy of Translation [CAT], 2020a, 2020b, 2020c). In this article, we explore the features of China English in the CAT’s translations related to COVID-19, representing a panoramic view of epidemic-stricken China.

## Literature Review

Some scholars have so far studied the characteristics of English as a first, second or foreign language used to report seminal global events, such as wars, PHEICs, terrorist attacks, and sports games. For example, McArthur (2003) analyzed the usage of Westerners’ language related to the 9/11 terrorist attacks; Fong (2009) explored the reasons for the English fever in China before and during the Beijing Olympics; Coleman (2016) discussed some Vietnam War slang expressions collected in a slang dictionary. Eagleton (2004) analyzed the features of English used by a Hong Kong-based newspaper during the SARS outbreak, focusing on use of some names, word play, heroic rhetoric, and especially metaphors such as “disease as war” and “disease as detection,” and claiming that these features are closely associated with Chinese culture. Both Crystal (2020) and Roig-Marín (2021) noted that blending is a major word-formation device in producing English neologisms related to COVID-19, and the former also mentioned coinage, abbreviation, and analogy, saying at his personal website that “I wouldn’t be surprised to find Covid regional dialects develop in the UK, USA, Australia, and so on. And what’s going on in other languages, I wonder?” (Crystal, 2020). Therefore, in some sense, this study is a response to David Crystal’s question.

It is of great importance to study English used in reports on big events, such as COVID-19, especially in countries and regions where English is used as a second or foreign language because such English has a great influence on English varieties, such as China English. As a major variety of world Englishes, China English, formerly labeled “Chinglish,” refers to “English with Chinese characteristics” (Pinkham, 2000, p. 1), and it generally results from “the use of Chinese words that have been translated literally into English” (Xu & Tian, 2017, p. 2). China English has been acknowledged and discussed by some scholars. For example, Eaves (2011) discussed the extensive use of English in China’s bilingual public signs, believing that “[w]ith so many learners there, it stands to reason that a variety of English peculiar to China would eventually develop, and there is much evidence to suggest that it has already begun.” Bolton and Graddol (2012) pointed out that there were about 400 million English learners in China in 2010, and “[t]he current popularity of English in China is unprecedented, and has been fueled by the recent political and social development of Chinese society.” Botha (2014) discussed the use of English in China’s universities which is “dramatically altering the linguistic nature of students’ educational as well as their personal lives”; Yiyang (2019) argued that it is necessary to use the term “China English” instead of the “pejoratively perceived ‘Chinese English’ or ‘Chinglish’,” holding the view that “’China English’ as a legitimate variety does exist”; Du et al. (2020) examined the Chinese graduate students’ use of China English in the United States to “transform an Inner Circle academic space into one in which different varieties of English can be an effective communicative resource.”

The production of China English is inseparable from the effects of Chinese language and culture. The Chinese language is characterized by parataxis in that logical relations between phrases, clauses, and sentences are implied in many cases, and various sentence parts such as subject and object may be omitted, which most often does not cause confusion or misunderstanding (Pan, 1997; Tian, 2013). Generally speaking, the subject or the object should be added in translating into English the Chinese sentence typology without a subject or an object. Repetition is another important feature of Chinese, and in many cases, it cannot be retained in translation from Chinese to English; otherwise, it would turn out to be a bad repetition. Admittedly, there are also repetitions of words in English, such as the opening sentence in Charles Dickens’ masterpiece *A Tale of Two Cities*: “It was the best of times, it was the worst of times, it was the age of wisdom, it was the age of foolishness, it was the epoch of belief, it was the epoch of incredulity. . ..” (Dickens, 2017, p. 1). However, what is important is that we should clearly know and keep in mind the similarities and differences in use of repetition between Chinese and English, and choose proper methods such as literal translation, free translation, omission, and substitution in Chinese-English translation (Tian, 2013, pp. 40–42).

It seems that scanty research has so far been carried out to explore the features of China English related to big events, such as the Beijing Olympics, the Shanghai World Expo, the SARS outbreak, and COVID-19, even though English was widely and frequently used by Chinese media to report these events, and the relevant English reports had a significant impact on the development trend of China English. Therefore, in the present study, we explore the features of China English related to the coronavirus outbreak, focusing on the CAT’s translations which were extensively used by Chinese media.

## Research Methodology

As a research institution of the CFLPA, the CAT was established in 2014. It focuses on research on the translation of major projects at the national level, particularly with respect to the Chinese way of development, China’s political thinking, and socialism with Chinese characteristics (People.cn, 2014). This determines the Chinese characteristics of the CAT’s translations. The Communist Party of China (CPC) and the Central Government made many guidelines for combating the coronavirus, and President Xi Jinping gave important talks on the COVID-19 prevention and control. Therefore, translation of these guidelines, talks, and the epidemic-related terminologies became a major project for the CAT which duly completed and released five groups of translations entitled *Key Words to Understand China: The Fight Against COVID-19* (henceforth “KWUC-FAC”) at its WeChat public platform by the middle of April 2020, and the first three groups in more than a dozen foreign languages were officially approved and released by the CFLPA via various media and channels, such as the website of the Translators Association of China (China Foreign Languages Publishing Administration [CFLPA], 2020).

The KWUC-FAC is a Chinese-English bilingual version which consists of six sections, including “Decisions by the Central Leadership,” “Anti-Epidemic Guidelines and Arrangements,” “Effective Measures,” “About COVID-19,” “International Aid,” and “Brave Fighters” (CAT, 2020a). In each section, the key words, phrases or sentences in the Chinese government documents are used as titles and followed by a detailed account of them. There are a total of 615 key words in the five groups in which 181 words in the first three groups had been authorized by the CFLPA. A comparative approach is taken in this study to compare Chinese originals with their English translations, and these translations are also compared with similar English expressions in Chinese and Western media in order to summarize the features of epidemic-related China English. Data for this project is taken from the websites or WeChat public platforms of the following sources: (1) China Academy of Translation; (2) *China Daily*; (3) Xinhuanet-English version; (4) *The New York Times*, *The Guardian*, *US Today*, *The Australian*, and *New Scientist*. There are 52,417 words/characters in the corpus of this study, the inclusion of the linguistic innovations into the sample is purely thematic, and the new verbal signs which occur once in the corpus are included into the sample. A few abbreviations are used in the case study for the convenience of discussion, including PY, WT, LT, and CT which, respectively, refer to Chinese Pinyin version, word-for-word translation, literal translation, and the CAT’s translation of Chinese examples. In the following sections, we analyze the linguistic, ideological, and cultural features of English used in the CAT’s translations from bilingual, translation, and bicultural perspectives.

## Linguistic Features

Translation can be defined as “the replacement of textual material in one language (SL) by equivalent textual material in another language (TL)” (Catford, 1965, p. 20). In other words, translation is a language transformation activity, and in some sense, translation features can be seen as linguistic features. The CAT’s translations are characterized by linguistic normativity, lexical redundancy, semantic explicitation, hypotactic conversion, omission of repetitions, and use of notes. The CAT used standard terminologies, such as “novel coronavirus pneumonia” and “COVID-19,” throughout all its translations after the National Health Commission of China and the World Health Organization (WHO) had officially released them. However, Western media often used nonstandard forms of these terminologies, such as “Covid-19” (The New York Times, 2020), “covid-19” (New Scientist, 2020), “new coronavirus” (Trilling, 2020), or even “China virus” (Peel & Robinson, 2020). Sometimes, lexical redundancy is seen in the translations. For example:

1    新增  确诊  病例

PY:  *xīnzēng  quèzhěn  bìnglì*

CT:  newly confirmed cases

(KWUC-FAC, 6 March 2020)

2  对  抗疫  医务  人员  保护、  关心、  爱护

PY:  *du  kàngyì  yīwù  rényuán  băohù    guānxīn  àihù*

CT:  to provide full protection and care to medical workers fighting against the epidemic

(KWUC-FAC, 25 March 2020)

3  打赢  疫情  防控   狙击战

PY:  *dăyíng  yìqíng  fángkòng   zŭjīzhàn*

CT:  to fight and win the battle against the epidemic

(KWUC-FAC, 13 March 2020)

In examples 1 and 2, the expressions “newly confirmed cases” and “fighting against the epidemic” are acceptable, but they are not as concise as “new infections” and “fight the coronavirus” used by Western media (The New York Times, 2020). In example 3, the word “打” (*dă*, “fight”) in the set phrase “打赢” (*dǎyíng*, “fight and win”) seems unnecessary and thus can be omitted because “win the battle” presupposes “fight the battle.” In Chinese, omission of some sentence part gives rise to obscurity in meaning or semantic implicitness which is an important feature of Chinese (Pan, 1997, pp. 203–204; Tian, 2013, p. 101), and in the translation from Chinese to English, the implied contextual meaning should be clearly represented via addition of words in order to facilitate target readers’ understanding. For example:

4  集中  拉网式    大  排查

PY:  *jízhōng  lāwăngshì  dà  páichá*

WT:  concentrated dragnet-style large screening

CT:  massive dragnet screening of potential virus carriers

(KWUC-FAC, 6 March 2020)

5  外防  输入、  内防   扩散

PY:  *wàifáng  shūrù,  nèifáng  kuòsàn*  WT  outside prevent entering, inside prevent spreading

CT:  preventing the coronavirus from entering and spreading within a region

(KWUC-FAC, 6 March 2020)

6  应收  尽收、  应治  尽治

PY:  *yīngshōu  jìnshōu,  yīngzhì  jìnzhì*  WT:  should admit all admitted, should treat all treated

CT:  admitting all suspected and confirmed cases for treatment

(KWUC-FAC, 25 March 2020)

In example 4, the Chinese original does not tell us who should carry out the screening work and what should be screened, and according to the context, the subject is “Wuhan City” (“武汉市,” *wŭhànshì*) and the object is “four groups of people” (“四类人员,” *sìlèi rényuán*) which include confirmed cases, suspected cases, febrile patients who cannot rule out the possibility of infection, and close contacts. Likewise, in examples 5 and 6, it is not clear what should be prevented from entering from outside and spreading inside, and who should be admitted and treated. The CAT’s translations illustrate that they are “coronavirus” and “suspected and confirmed cases.” Specifically, the context indicates that as for “外防输入、内防扩散,” the subject is “the community” (“社区,” *shèqū*) and the object is “imported cases” (“输入性病例,” *shūrùxìng bìnglì*) and “the outbreak” (“疫情,” *yìqíng*); as for “应收尽收、应治尽治,” the subject is “the hospitals” (“医院,” *yīyuàn*) and the object is “confirmed and suspected cases” (“确诊和疑似病例,” *quèzhěn hé yísì bìnglì*). Therefore, the implied context-bound meaning should be clarified by adding proper words in translating in order to help the international community to understand China’s official guidelines on the epidemic control completely. Parataxis is another feature of Chinese in which phrases and clauses are often juxtaposed without the use of connectors, while English is a hypotactic language in which phrases and clauses are joined with conjunctions or prepositions (Pan, 1997, p. 203; Tian, 2013, p. 19). Therefore, conversion of parataxis to hypotaxis is necessary in rendering Chinese paratactic expressions. For example:

7  集中  患者、  集中  专家、  集中    资源、 

PY:  *jízhōng  huànzhě,  jízhōng  zhuānjiā  jízhōng  zīyuán*,

集中  救治

*jízhōng  jiùzhì*

WT:  concentrate patients, concentrate experts, concentrate resources, concentrate treatment

CT:  treating the infected in dedicated facilities by senior medical professionals from all over the country and with all necessary resources

(KWUC-FAC, 13 March 2020)

In example 7, the Chinese original is a typical paratactic sentence in which four clauses are put together without using a connector in between. However, in the CAT’s translation, the Chinese paratactic sentence is transformed into a hypotactic one in the English version via the use of such connectors as “in,” “by,” “from,” “and,” and “with.” Moreover, the word “集中” (*jízhōng*, “concentrate”) is used four times in the original. This lexical repetition is a striking feature of Chinese which can be viewed as a special form of hypotaxis (Pan, 1997, p. 350, p. 374), as best illustrated in the Song-Dynasty poet Li Qingzhao’s verse line “寻寻觅觅, 冷冷清清, 凄凄惨惨戚戚” (*xúnxún mìmì, lěnglěng qīngqīng, qīqī căncăn qīqī*, “seek, seek, search, search; cold, cold, clear, clear; sad, sad, wretched, wretched, miserable, miserable”). But the word “集中” is omitted in the translation, and its meaning is implied by “dedicated” and “all.” Let us look at one more example:

8  早  发现、  早  报告、  早  隔离、  早  治疗

PY:  *zăo  fāxiàn,  zăo  bàogào,  zăo gélí,    zăo  zhìliáo*

WT:  early discovery, early report, early isolation, early treatment

CT:  early detection, reporting, isolation and treatment

(KWUC-FAC, 13 March 2020)

In example 8, the word “早” (*zăo*) is used four times in the original, while its literal rendering “early” is used only once in the English version because repetition should, in many cases, be avoided in English via ellipsis or substitution (Tian, 2013, p. 41). As for Chinese-specific words and expressions, occasional use of notes is seen in the CAT’s translations. For example:

9  最美  逆行  者

PY:   *zuìměi  nixing  zhě*

WT:   most beautiful reverse walker

CT:   heroes in harm’s way (the brave, unhesitating rescuers who rush to the epicenter of the virus)

(KWUC-FAC, 25 March 2020)

10  对口  支援

PY: *  duìkŏu  zhīyuán*

CT:  pairing assistance (a national strategy in China for one province or a major city to provide assistance to a designated region in need of help)

(KWUC-FAC, 25 March 2020)

The CAT’s English version in example 9 is a free translation for the original, while the translation in example 10 is a literal rendering. Without the notes, it is almost impossible for target readers to understand the exact meaning of the translations “heroes in harm’s way” and “pairing assistance,” even though they know that these expressions are closely related to the COVID-19 outbreak.

## Ideological Features

In containing the coronavirus spread, great achievements had been achieved in China under the leadership of the CPC and the Central Government whose efforts were acknowledged by the international community. Just as the WHO pointed out, “China has rolled out perhaps the most ambitious, agile and aggressive disease containment effort in history” (De Ceukelaire & Bodini, 2020). The scientific policy-making and effective decisions of the central leadership played a crucial role in defeating the coronavirus. In the anti-epidemic guidelines proposed by the CPC Central Committee and the State Council, especially President Xi’s talks, there are a large number of powerful metaphors, most of which are related to battles and wars, such as “战疫” (*zhànyì*, “virus-fighting”), “奋战” (*fènzhàn*, “bravely fight”), “作战” (*zuòzhàn*, “fight”), “战斗” (*zhàndòu*, “combat”), “备战” (*bèizhàn*, “prepare for war”), “战胜” (*zhànshèng*, “fight and win”), “打赢” (*dăyíng*, “fight and win”), “阻击战” (*zŭjīzhàn*, “blocking action”), “战斗力” (*zhàndòulì*, “fighting capacity”), “攻坚战” (*gōngjiānzhàn*, “tough fighting”), “总体战” (*zŏngtĭzhàn*, “total warfare”), “人民战争” (*rénmín zhànzhēng*, “people’s war”), “战线” (*zhànxiàn*, “battle line”), “前线” (*qiánxiàn*, “front line”), “防线” (*fángxiàn*, “line of defense”), “军令状” (*jūnlìngzhuàng*, “military order”), “堡垒” (*băolěi*, “fortress”), and “牺牲” (*xīshēng*, “sacrifice”). The use of war metaphors in the Chinese discourse of battling the coronavirus reflects the militarism of the Chinese people (Tian & Wang, 2017, p. 127), and it can build up their confidence in stopping the epidemic. However, many of these war metaphors are lost in the CAT’s translations. For example:

11  打赢  疫情  防控   的  人民  战争、  总体战、 

PY:  *dăyíng  yìqíng  fángkòng   de  rénmín  zhànzhēng,  zŏngtĭzhàn*,  

阻击战

*zŭjīzhàn*

WT:  fight win epidemic prevention control people’s war, total warfare, tough fighting

CT:   to win the nation’s war on the epidemic

(KWUC-FAC, 6 March 2020)

12  这  是  动员令,  也是  宣言书,  更是 

PY:   *zhè  shì  dòngyuánling,  yěshì  xuānyánshū,  gèngshì*
 

军令状。

*jūnlìngzhuàng.*

WT:   This is mobilization order, also is manifesto, moreover is military order.

CT:   This is a mobilization and an order.

(KWUC-FAC, 6 March 2020)

Three war-related expressions are used in the original in example 11, including “人民战争” (*rénmín zhànzhēng*, “people’s war”), “总体战” (*zŏngtĭzhàn*, “total warfare”), and “阻击战” (*zŭjīzhàn*, “tough fighting”), but none of them is retained in the translation. Instead, the general expression “the nation’s war” is used to cover and represent their meanings. Similarly, the powerful expressions of “动员令” (*dòngyuánling*, “mobilization order”), “宣言书” (*xuānyánshū*, “manifesto”), and “军令状” (*jūnlìngzhuàng*, “military order”) are not faithfully represented in the English version in example 12 because “令” (*lìng*, “order”) in “动员令,” “宣言书,” and “军令” (*jūnlìng*, “military”) in “军令状” are not translated, resulting in the weakening of the expected effectiveness of publicity. Other types of metaphor are also seen in Xi’s talks. For example:

13  生命    重于  泰山。

PY: *  shēngmìng  zhòngyú  tàishān.*

LT:   Life is weightier than Mount Tai.

CT:   Saving lives is of paramount importance.

(KWUC-FAC, 13 March 2020)

14  疫情  就是  命令，  防控  就是  责任。

PY:   *yìqíng  jiùshì  mìngling,  fángkòng  jiùshì  zérèn.*

LT:   COVID-19 is the order; its prevention and control is the responsibility.

CT:   Go where there is epidemic, fight it till it perishes.

(CAT, 6 March 2020)

In the Chinese sentences in examples 13 and 14, people’s life is compared to Mount Tai, and the epidemic to a military order, which emphasized on the significance of safeguarding the people’s lives and controlling the virus spread. The belief contained in example 13 that the life of all people is of equal importance constitutes a sharp contrast to some Western leaders’ philosophy of herd immunity and natural elimination which put elderly people at great risk (The New York Times, 2020). In the CAT’s translations, the free translation strategy is adopted in order to help target readers to understand the original well. As a result, the metaphors and the characteristics of the Chinese expressions have got lost. It is noteworthy that “Go where there is epidemic, fight it till it perishes” in example 14 is of paratactic language because no connector is used between the two clauses, thus representing the parataxis in the original. As a major feature of Chinese, lexical repetition should often be avoided in the translation from Chinese to English, but it can sometimes be kept in translating in order to achieve emphasis on a certain meaning or powerfulness in expression. For example:

15  武汉  胜  则  湖北  胜,  湖北    胜  则  全国  

PY: *  wŭhàn  shèng  zé  húběi  shèng  húběi    shèng  zé  quánguó*
 

胜。

*shèng.*

CT:   If Wuhan wins, Hubei wins. If Hubei wins, the whole country wins.

(KWUC-FAC, 25 March 2020)

COVID-19 first broke out in Wuhan, Hubei’s capital city in January 2020, and Hubei was the hardest-hit province in China. Therefore, the central task of the whole country focused on the epidemic control in Wuhan and Hubei. The English version in example 15 is a literal rendering, and by repeating the word “胜” (*shèng*, “win”), President Xi clearly pointed out the key to battling the virus by saying that China would eventually win the war of battling the epidemic if Wuhan and Hubei could successfully control the virus, and he called on the whole nation to provide assistance to them. This example shows that there are also good repetitions of words in English, just like the opening sentence in Charles Dickens’ *A Tale of Two Cities*. It should be noted that unique political language was often seen in the CPC’s guidelines, and some expressions excerpted from Xi’s talks had become slogans for all Party members and cadres in combating the coronavirus. For example:

16  让  党旗  在  防控  疫情  斗争    第一线

PY:   *ràng  dăngqí  zài  fángkòng  yìqíng  dòuzhēng  dìyīxiàn*

高高  飘扬。

*gāogāo piāoyáng.*

CT:   Let the Party flag fly high on the front line of the anti-epidemic war.

(KWUC-FAC, 25 March 2020)

17   一个  党员   就是  一面  旗帜,  一个  支部  就是  

PY:   *yīgè  dăngyuán   jiùshì  yīmiàn  qízhì,  yīgè  zhībù  jiùshì*

一座  堡垒。

*yīzuò  băolěi.*

CAT:  A Party member is like a flag and a Party branch is like a fortress.

(KWUC-FAC, 13 March 2020)

The political slogans in examples 16 and 17 have special connotations in the context of the CPC leadership. Confronted with the epidemic crisis, President Xi asked all Party members and Party organizations at all levels to take the lead to bring into full play their abilities by going deep into grass-roots communities and villages to fight the virus. This was clearly illustrated in the catchphrase “党员干部下沉” (*dăngyuán gànbù xiàchén*) during the outbreak, which means the deep community engagement of all Party members and cadres in the epidemic prevention and control (CAT, 2020c). Finally, the shift in emotional coloring is sometimes seen in translating commendatory terms. For example, as a commendatory word, “殉职” (*xùnzhí*, “die at one’s post”) is rendered as the neutral word “die”; “崇高” (*chónggāo*, “lofty”) in “崇高的敬意” (*chónggāo de jìngyì*, “lofty respect”) is also a commendatory word but omitted, resulting in the weakening of the original commendatory coloring (CAT,2020c).

## Cultural Features

Due to the lockdown and self-quarantine, COVID-19 had a great impact on China’s politics, economy, culture, healthcare, education, travel, and daily life. This impact was reflected in the CAT’s translations. For example:

18  火神山    医院

PY:   *huŏshénshān  yīyuàn*

CT:   Huoshenshan Hospital

(KWUC-FAC, 25 March 2020)

19  雷神山    医院

PY:   *léishénshān  yīyuàn*

CT:   Leishenshan Hospital

(KWUC-FAC, 25 March 2020)

20  中西医    并用

PY:   *zhōngxīyī  bìngyòng*

CT:   combined use of TCM and Western medicine

(KWUC-FAC, 13 March 2020)

Wuhan was the epicenter of the epidemic in China and the first city in lockdown, with more than 50,000 infections. As the number of infected people skyrocketed, the hospitals available in Wuhan were unable to tackle confirmed cases. As a result, two big hospitals were built within 20 days, a “Chinese speed” in times of urgency, and they were named “Huoshenshan Hospital” and “Leishenshan Hospital.” As illustrated in examples 18 and 19, “Huoshenshan” and “Leishenshan” are the Chinese Pinyin versions or transliterations of “火神山” and “雷神山” whose literal meanings are “Fire God Mountain” and “Thunder God Mountain.” It was believed that the Fire God and the Thunder God could help the Chinese to stop the epidemic because they take control of fire and lightning which can kill the virus (Zhang, 2020). Moreover, many “makeshift hospitals” or “temporary treatment centers” (“方舱医院,” *fāngcāng yīyuàn*) were built to deal with suspected or mild cases (CAT, 2020a), and it can be argued that the Chinese original “方舱” (*fāngcāng*, “shelter”) in the name of hospitals of this category might allude to Noah’s ark which is translated into Chinese as “诺亚方舟” (*nuòyà fāngzhōu*). As the saying goes, God help those who help themselves. The self-reliance of the Chinese nation played a decisive role in controlling COVID-19. As seen in example 20, in the treatment of infected people, traditional Chinese medicine (TCM) and Western medicine were combined because they have their own advantages, and their combined use “had achieved good effects during China’s fight against SARS in 2003” (CAT, 2020c). During the period of lockdown, hundreds of millions of people had been placed in some form of isolation in order to contain the virus spread (Qin, 2020). The supply and transportation of daily necessities became an essential issue for millions of people, and the CPC Central Committee proposed the relevant guidelines. For example:

21    “米袋子”   省长    责任制  和  “菜篮子”   PY:   “mĭdàizĭ”   shěngzhăng  zérènzhì  hé  “càilánzĭ”

    市长  负责制

shìzhăng  fùzézhì

CT:   systems of holding provincial governors accountable for grain supplies and city mayors for daily food supplies

(KWUC-FAC, 25 March 2020)

22    解决  好  生活   必需品供应    的  

PY:   jiějué  hăo  shēnghuó   bìxūpĭn  gòngyīng  de

“最后  一  公里”  问题

“zuìhòu  yī  gōnglĭ”   wèntí

CT:   to ensure the “last kilometer” delivery of daily necessities

(KWUC-FAC, 25 March 2020)

23    武汉  快递  小哥  汪勇：  组织  志愿者

PY:   wǔhàn  kuàidì  xiăogē  wāng  yŏng:  zŭzhī  zhìyuànzhě

接送  医护  人员

jiēsòng yīhù rényuán

CT:   Wang Yong: a courier volunteer

(KWUC-FAC, 13 March 2020)

On behalf of the CPC Central Committee, Vice Premier Sun Chunlan, together with the Central Guiding Team, arrived in Wuhan on January 27, 2020, and found that daily necessities could not be sent to residents fast and smoothly (CAT, 2020a). Therefore, it was proposed that the governors and city mayors should take full responsibility for food supplies, such as Chinese cabbage, pakchoi seedlings, and musang king durians (Cheng, 2020), and ensure the “last kilometer” smooth delivery of food and other daily necessities. With the help of tens of thousands of community cadres and courier volunteers, such as Wang Yong, ample and timely daily supplies were guaranteed, as indicated in examples 21, 22, and 23. As for quarantined city dwellers who were unable to do the cooking by themselves, the meals were brought to them by thousands of “deliverymen” (“快递小哥,” *kuàidì xiǎogē*) who were praised by Western media because they took the risk of losing their lives to do so (Wang, 2020). In the field of education, the COVID-19 outbreak had a great impact on students at all levels, including college graduates. For example:

24  高校  学生  毕业、  招聘、  考录

PY:   *gāoxiào  xuéshēng  bìyè,  zhāopìn,  kăolù*

CT:   graduation and job placement of college students

(KWUC-FAC, 13 March 2020)

The outbreak forced China’s Ministry of Education (MOE) to postpone the commencement of the spring semester, and launch the “Home Study Initiative” (“停课不停学,” *tíngkè bú tíngxué*) (MOE, 2020b). Based on the Open University of China, the MOE provided free online courses, including quality MOOCs (i.e., massive open online courses), to support students at all educational levels to continue studying at home. As for college students’ “graduation and job placement” as indicated in example 24, the services of “online campus recruitment,” “online interview,” and “signing an employment contract online” were provided for them by the MOE (Helen, 2020a). After the COVID-19 broke out, the international community lent their valuable support to China in the form of materials and regards. For example:

25  山川     异域,   风月   同天

PY:   *shānchuān yìyù,   fēngyuè   tóngtiān*

WT:   Mountain river different region, wind moon same sky

CT:   Miles apart, but close at heart

(KWUC-FAC, 25 March 2020)

The Tokyo-based Japan Youth Development Association donated 20,000 masks to Hubei, on the boxes of which was written the Chinese verse line “山川异域, 风月同天” (“Lands apart, sky shared”), and this powerful poetic message had a very positive response on Chinese social media (*China Daily*, 27 February 2020). As a form of gratitude, there appeared many English versions of the verse line by the Chinese, such as “Miles apart, but close at heart” in example 25 and “Rivers low, mountains high; the same moon in the sky” by Professor Zhao Yanchun, the translator of the Chinese classic *Three Character Classic*.

## Discussion and Implications

Admittedly, the CAT’s translations which are, in some cases, characterized by China English played an important role in policy publicity and information dissemination during the outbreak. It seems unnecessary for China English to get close to Standard English in order to achieve idiomatic expression, for the very purpose of maintaining and enriching it as a major English variety. For example, the CAT’s rendering of “疫情就是命令, 防控就责任” is “Go where there is epidemic, fight it till it perishes,” which should be improved as something like “Go where there is epidemic, and fight it till it perishes” or “Go where there is epidemic; fight it till it perishes” according to English usage. But we think that as a paratactic sentence, the translation is acceptable because it is smooth and clear in meaning. The translation of the same sentence by the Xinhua News Agency is “Life is of paramount importance. When an epidemic breaks out, a command is issued. It is our responsibility to prevent and control it” (Huaxin, 2020). We do not think that it is suitable for the original as a powerful, concise slogan, even though it is completely in accordance with English grammar. The literal rendering of the sentence as “COVID-19 is the order; its prevention and control is the responsibility” can better illustrate the characteristic of Chinese language and thinking.

Lexical repetition can be retained in translating in most cases in order to achieve semantic emphasis and represent Chinese characteristics. For instance, the CAT’s translation of “early detection, reporting, isolation and treatment” for “早发现, 早报告, 早隔离, 早治疗” can be modified as “early detection, early reporting, early isolation, early treatment,” which can better show China’s fast responses to the coronavirus. War metaphors as an important feature in the epidemic-related Chinese discourse can also be maintained, as illustrated in “people’s war, total warfare, tough fighting” for “人民战争, 总体战, 阻击战” which is better than the CAT’s generalization of “the nation’s war.” Similarly, the CAT’s generalization of “宁可十防九空, 不可失防万一” (*níngkě shífáng jiŭkōng, búkě shīfáng wànyī*) as “China will do everything possible to combat it” (CAT, 2020a) is not as good as the literal rendering of “Better be in vain in nine out of ten prevention efforts than lose the defense line in one out of ten thousand efforts.” Although a number of translation methods are employed in the CAT’s translations of the 25 examples above with a varying use frequency, such as annotation (2 times), free translation (2 times), addition (3 times), omission (8 times), and literal translation/transliteration (10 times), it can be argued that the methods of literal translation and transliteration can best keep Chinese characteristics in rendering Chinese-specific words and expressions, such as “fight and win” for “打赢,” “actively win” for “积极争取,” and “Huoshenshan Hospital” for “火神山医院.” It is not advisable to achieve clarity in meaning and brevity in expression at the cost of losing Chinese uniqueness in foreign publicity.

The use of war-linked phrases is a striking feature in the Chinese government documents related to the prevention and control of COVID-19, and it was also seen in Hong Kong’s newspaper reports on combating the SARS (Eagleton, 2004). In some cases, the CAT’s translations use omission or generalization to reduce the negative connotations underlying the phrases. This translation strategy taken by the CAT is aimed at achieving the very purpose of the Chinese government’s foreign publicity, and the translation principle followed by the CAT is the three kinds of “closeness” proposed by Huang Youyi, the former vice director of the CFLPA, namely closeness to China’s developmental reality, closeness to foreign audiences’ demand for information on China, and closeness to their thinking habit (Liu, 2021, p. 74).

The 2020 campaign of fighting and containing the virus around the world seems to indicate that all the countries and regions on Earth constitute “a community with a shared future” (“命运共同体,” *mìngyùn gòngtóngtǐ*), a world peace and security-oriented concept proposed by China (CAT, 2020b). The pandemic has proved that all of us live in one and the same world, and only when the whole world should be united as one, could the virus be wiped out. However, the ideological confrontation seems to continue to exist between China and the West. The fact that COVID-19 first broke out in China made some Westerners call it “Chinese virus” or “China virus,” as used in Western media’s headlines, such as “Schools isolate students in danger of China virus” (*The Australian*, 28 January 2020). The WHO named the new virus “COVID-19” for the very purpose of preventing the use of such prejudiced terms which are related to “a geographical location, an animal, an individual or a group of people” (Ma & Tang, 2020). Moreover, Westerners’ antagonism toward Chinese or Asian citizens due to the virus was sometimes seen in the West during the outbreak. For example, a student from Singapore was attacked by a group of men in London, one of whom shouted, “I don’t want your coronavirus in my country” (The New York Times, 2020).

In fact, coordination of adequate collective responses is of paramount importance within and across countries and regions in coping with PHEICs, such as COVID-19 (De Ceukelaire & Bodini, 2020). And intercultural communication and solidarity can mobilize support from all walks of life. Both Chinese and Western leaders encouraged their peoples to combat the virus spread (see Table 1).

**Table 1.** Words of encouragement by Xi, Trump and Johnson.

Xi	Go where there is epidemic, fight it till it perishes. We are determined to fight and win the battle against the epidemic by mobilizing all resources to control the spread of the virus. If Wuhan wins, Hubei wins. If Hubei wins, the whole country wins. (CAT, 2020b)
Trump	No nation is more prepared or more resilient than the United States. We have the best economy, the most advanced healthcare, and the most talented doctors, scientists, and researchers anywhere in the world [. . .] As history has proven time and time again, Americans always rise to the challenge and overcome adversity. Our future remains brighter than anyone can imagine. Acting with compassion and love, we will heal the sick, care for those in need, help our fellow citizens, and emerge from this challenge stronger and more unified than ever before. (US Today, 2020)
Johnson	We will get through this, this country will get through this epidemic, just as it has got through many tougher experiences before if we look out for each other and commit wholeheartedly to a full national effort. (The Guardian, 12 March 2020)

In President Xi’s virus-fighting talks, he called on millions of Party members and cadres to be actively committed to community engagement in preventing and controlling the virus spread. He tried to heighten the confidence of the Chinese people by promising to mobilize all resources of the country, and pointed out the central task of stopping the epidemic, namely, Wuhan’s victory over the outbreak. In his televised speech, the US President Donald Trump attempted to build up the confidence of Americans by flaunting the US strength in economy, healthcare, and science. Like President Trump, Britain’s Prime Minister Boris Johnson used the indirect general expression of “victories in history” to strengthen the belief of the British in getting through the ordeal. In fact, it is not a single nation-state but all humans that were in the same boat in combating COVID-19 as a huge global public health challenge.

During the extended lockdown, millions of families were forced to live in confined spaces in Wuhan and other cities in Hubei. Many problems, such as boredom and physical abuse, took place in these families (Cooper & Marshall, 2020; Qin, 2020). Moreover, it was difficult for them to find help because it was almost impossible for them to leave the house. One solution to fighting the boredom was to attend master classes posted by Shanghai-based quarantined musicians on their WeChat page, and watch the players practicing at home and playlists (Cooper & Marshall, 2020). A similar case in point is Bahargul Toleheng, a medical worker who came from Xinjiang Uygur Autonomous Region to provide assistance to Hubei (CAT, 2020c). When she learned that some patients were feeling down in the dumps, Bahargul volunteered to dance in the treatment center as a way to cheer them up. She performed a traditional Kazak dance and received a rousing ovation from the patients. On the one hand, these examples indicate that psychological issues and family violence with quarantined people and patients in times of PHEICs should be studied by the government and academia; on the other hand, the voluntary musicians’ selfless endeavors provided some implications for solving the problems. And we cannot forget poetry in that it seems to “hold a special power to comfort and connect” (Helen, 2020b). The verse line of “山川异域, 风月同天” as a message of heartfelt regards sent by Japanese people alluded to a significant moment in the history of cultural exchanges between the two countries. It comes from a seventh-century poem by Prince Nagaya, a politician who lived in Japan’s Nara period (710-794), with the title being *Embroidered on Kasaya Robes for Good Karma*. “As the title suggests, the poem was embroidered on 1,000 Buddhist robes which Prince Nagaya had made and sent to the Tang court in China, as a token of his invitation for Chinese Buddhist monks to visit Japan; inspired by the Prince’s poem, Jianzhen, the renowned traveler and spiritual leader in the Tang Dynasty (618-907), decided to go on a voyage to Japan; after six attempts and the loss of his eyesight, Jianzhen finally arrived, and made a significant contribution to the spread of Buddhism in Japan” (*China Daily*, 27 February 2020). The story behind the verse line invoked both shared history and mutual appreciation for ancient poetry in Chinese. Many Chinese people were touched by the poetic expression of support from the Japanese during the COVID-19 outbreak.

## Conclusion

English was used widely and frequently in Chinese media during the COVID-19 outbreak for daily precautions, policy publicity, and information dissemination, and the CAT’s translations provided the media with importance reference for the use of English. China English was outstanding with respect to translating some Chinese-specific words and expressions in the translations, and it generally resulted from the method of literal rendering. With this method, some of the CAT’s translations can be improved with more Chinese characteristics if we take linguistic acceptability into consideration at the same time. In some sense, translation, especially literal translation, is an important way to develop and enrich China English. As for ideological publicity, by virtue of lexical repetitions and use of war metaphors, the greatest importance was attached to people’s lives in President Xi’s talks which highlighted high efficiency and strategic rationality in combating the virus. Although there were some problems during the period of lockdown, such as boredom and physical abuse, poetic message and musical power brought comfort and warmth to quarantined people in cold China in the late winter and early spring of 2020.

## Notes

1. For detail, see the report *WHO-convened Global Study of Origins of SARS-CoV-2: China Part* on the website of the WHO (https://www.who.int/health-topics/coronavirus/origins-of-the-virus).
